# Durable effectiveness and safety of hybrid ablation versus catheter ablation: 2-year results from the randomized CEASE-AF trial^†^

**DOI:** 10.1093/ejcts/ezaf146

**Published:** 2025-07-25

**Authors:** Nicolas Doll, Timo Weimar, Dariusz A Kosior, Alan Bulava, Ales Mokracek, Gerold Mönnig, Jonathan Sahu, Steven Hunter, Maurits Wijffels, Bart van Putte, Norman Rüb, Petr Nemec, Tomas Ostrizek, Erik Fransen, Piotr Suwalski

**Affiliations:** Departement of Cardiac Surgery, Schüchtermann-Klinik, Bad Rothenfelde, Germany; Eberhard Karls University, School of Medicine, Tuebingen, Germany; National Medical Institute of the Ministry of Interior and Administration, Centre of Postgraduate Medical Education, Warsaw, Poland; Mossakowski Medical Research Institute, Polish Academy of Sciences, Warsaw, Poland; Department of Cardiology, Ceske Budejovice Hospital, Czech Republic; Faculty of Health and Social Sciences, University of South Bohemia in Ceske Budejovice, Czech Republic; Faculty of Health and Social Sciences, University of South Bohemia in Ceske Budejovice, Czech Republic; Departement of Cardiac Surgery, Ceske Budejovice Hospital, Ceske Budejovice, Czech Republic; Department of Cardiology, Schüchtermann-Klinik, Bad Rothenfelde, Germany; Departement of Electrophysiology, Northern General Hospital, Sheffield, UK; Departement of Cardiac Surgery, Northern General Hospital, Sheffield, UK; Departement of Electrophysiology, St Antonius Hospital, Nieuwegein, The Netherlands; Departement of Cardiac Surgery, St Antonius Hospital, Nieuwegein, The Netherlands; Departement of Electrophysiology, RKH Klinikum Ludwigsburg, Ludwigsburg, Germany; Departement of Cardiac Surgery, Center of Cardiovascular Surgery and Transplantation, Brno, Czech Republic; Faculty of Medicine, Masaryk University, Brno, Czech Republic; Departement of Electrophysiology, Center of Cardiovascular Surgery and Transplantation, Brno, Czech Republic; AtriCure Europe B.V., Amsterdam, The Netherlands; National Medical Institute of the Ministry of Interior and Administration, Centre of Postgraduate Medical Education, Warsaw, Poland

**Keywords:** Atrial fibrillation, Hybrid ablation, Catheter ablation, Left atrial appendage, Surgical ablation

## Abstract

**OBJECTIVES:**

The CEASE-AF trial demonstrated that epicardial-endocardial hybrid ablation (HA) had superior effectiveness compared to endocardial catheter ablation (CA) for non-paroxysmal atrial fibrillation (AF), without significantly increasing major complications during a 12-month period. Most contemporary AF ablation trials have not evaluated durability beyond 12 months. Therefore, 24-month effectiveness and safety of HA and CA are compared.

**METHODS:**

CEASE-AF is a prospective, multicentre, randomized trial. Patients 18–75 years of age with symptomatic, drug refractory persistent AF and left atrial diameter >4.0 cm or long-standing persistent AF were randomized 2:1 to HA (posterior wall and pulmonary vein isolation with left atrial appendage exclusion) or CA (pulmonary vein isolation). Secondary effectiveness was freedom from AF/atrial flutter/atrial tachycardia off class I/III anti-arrhythmic drugs except for those who previously failed at doses not exceeding those previously failed through a 24-month follow-up period. Major complications and reinterventions were evaluated.

**RESULTS:**

The intention-to-treat population was 102 patients with HA and 52 patients with CA. Seventy-five percent were male, 80.5% had persistent AF and 19.5% had long-standing persistent AF, with a mean age of 60.7 ± 7.9 years. Effectiveness for 24 months was 66.3% (63/95) with HA and 33.3% (17/51) with CA [absolute difference 33.0% (95% confidence interval 14.3%, 48.3%; *P* < 0.001)]. Major complication rates were 10.8% (11/102) with HA and 9.6% (5/52) with CA (*P* = 1.0), and fewer patients had reinterventions after HA than CA [18.9% (18/95) vs 52.9% (27/51), *P* < 0.001].

**CONCLUSIONS:**

CEASE-AF demonstrated that the 32.4% absolute benefit of HA over CA for 12 months was durable for 24 months at 33% with continued similar safety rates and fewer reinterventions after HA (funded by AtriCure, Inc.; NCT02695277).

**Clinicaltrials.gov registration:**

NCT02695277

## Graphical abstract

**Figure:**
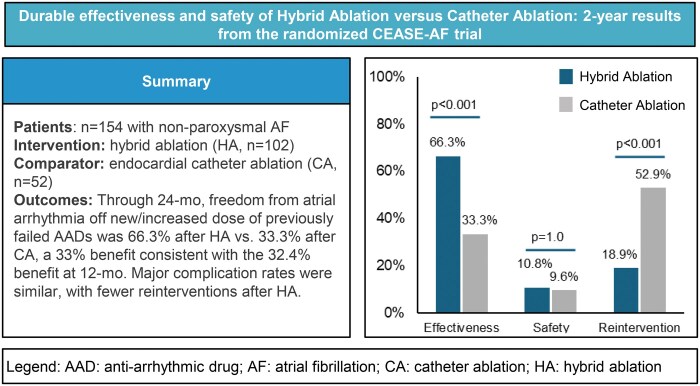

## INTRODUCTION

Atrial fibrillation (AF) is a progressive cardiomyopathy that was estimated to affect approximately 50 million people worldwide as of 2020 [1]. It can impair patient quality of life due to debilitating symptoms and confers substantially increased risks of stroke, heart failure and death. Early rhythm control for AF has been shown to be superior to rate control [2]. Clinical trials utilizing radiofrequency (RF), cryothermal and pulsed field endocardial ablation catheters to isolate the pulmonary veins (PVs) have reported rhythm control rates of 70–75% for 12 months in patients with paroxysmal AF [3–5]. However, in non-paroxysmal AF, endocardial ablation has proven more challenging, with trials reporting from 55% to 74% 1-year effectiveness even with advanced catheter technology and an aggregated historical success rate of 48% across more than 15 000 patients [6–11]. Additive endocardial ablation strategies to target non-pulmonary vein (PV) substrate, such as the left atrial posterior wall, have not shown a consistent rhythm control benefit over PV isolation (PVI) [12, 13]. In addition, published data on the durability of endocardial ablation as a rhythm control strategy in persistent and long-standing persistent AF are currently lacking. Most reported mid- and long-term effectiveness is from single centres [14, 15].

Hybrid ablation is an epicardial-endocardial ablation strategy, involving a heart team of electrophysiologists and surgeons, that has been evaluated in several clinical trials [16–18] and was included in the 2023–2024 AF treatment guidelines led by the American College of Cardiology, the European Heart Rhythm Association and the European Society of Cardiology [1, 19, 20]. CEASE-AF is the largest randomized controlled trial to date to compare a hybrid ablation (HA) strategy to an endocardial catheter ablation (CA) strategy, which showed superior effectiveness of HA and comparable safety over 12 months [17]. In this study, we report the 24-month outcomes of the CEASE-AF trial including effectiveness, safety and repeat interventions.

## PATIENTS AND METHODS

CEASE-AF (NCT02695277) is a prospective, multicentre, 2:1 randomized controlled trial carried out at 9 centres in Germany, the Netherlands, the United Kingdom, the Czech Republic and Poland. The trial was performed in accordance with the Declarations of Helsinki and BS EN ISO 14155: 2011. Ethics committee or institutional review board approval of the trial was obtained, and written informed consent was collected from each patient. A contract research organization (Cardialysis, Rotterdam, Netherlands) was responsible for data management, monitoring and analysis. A second contract research organization (Banook Group, Nancy, France) performed blinded review of electrocardiogram and Holter monitor recordings. The trial sponsor was AtriCure B.V. (Amsterdam, Netherlands).

The trial objective was to evaluate the efficacy and safety of 2 interventional approaches (HA vs standard CA) in preventing the recurrence of AF in symptomatic, drug refractory patients with advanced AF. The trial design and 12-month effectiveness and safety results were published previously [17].

Inclusion criteria were age 18–75 years and having symptomatic, drug-refractory persistent AF with an enlarged left atrium (diameter > 4.0 cm) or long-standing persistent AF. Key exclusion criteria were history of prior ablation, paroxysmal AF, long-standing persistent AF >10 years, AF secondary to electrolyte imbalance, thyroid disease or other reversible or non-cardiovascular cause, contraindication for catheter or epicardial ablation and/or the need for concomitant cardiac surgery. The HA arm consisted of a first stage epicardial procedure to, at minimum, isolate the PVs and create a box around the posterior left atrium with left atrial appendage exclusion (LAAE). Between 90 and 180 days after the index procedure, the endocardial ablation stage of the hybrid procedure was performed to address gaps to ensure PV and box lesion isolation. The CA arm received an index endocardial RF CA to, at minimum, isolate the PVs. Between 90 and 180 days post-index procedure, a repeat CA could be performed as clinically necessary. In both arms, additional lesions were permitted per institutional standard practice and were detailed in the previous publication [17].

Patients were followed up at 3 and 6 months (after the index procedure) and then at 6, 12 and 24 months after T0 (index procedure plus 6 months). Rhythm assessments were performed at scheduled visits during 48-h Holter monitoring, and symptom-driven rhythm monitoring was evaluated by a 12-lead electrocardiogram of at least 30 s at unscheduled visits. Medications and adverse events were also evaluated at each scheduled visit. Final analysis of the 36-month data is ongoing.

The primary effectiveness end point, as previously reported, was freedom from documented AF/atrial flutter (AFL)/atrial tachycardia (AT) episodes >30 s over a 12-month follow-up period, in the absence of a class I or III anti-arrhythmic drug (AAD) (with the exception of previously failed AADs at doses not exceeding those previously failed) [17]. A failure of the primary effectiveness end point included any documented AF/AFL/AT lasting >30 s; any previously failed class I or III AAD administered at a dose higher than baseline; any newly introduced class I or III AAD usage; a direct current cardioversion for AF/AFL/AT or any ablation intervention occurring during the 12-month follow-up visit, respectively. The present analysis is focused on secondary effectiveness over the 24-month post-T0, defined as freedom from documented AF/AFL/AT episodes >30 s over 24 months of follow-up, in the absence of class I or III AADs (with the exception of previously failed AADs at doses not exceeding those previously failed).

The safety end point consists of composite major complications during follow-up, comparing cumulative complication rates occurring during any procedure in the 2 study arms. Major complications are defined as death, stroke, transient ischaemic attack, myocardial infarction in the context of AF ablation, pericarditis, bleeding, wound infection, atrio-oesophageal fistula, oesophageal injury, permanent phrenic nerve paralysis, a permanent pacemaker implant as a direct result of injury to the specialized conduction system (sinoatrial or atrioventricular node), PV stenosis of >70%, cardiac tamponade/cardiac perforation, empyema, wound infection at the surgical site or puncture sites requiring reoperation for wound debridement, vascular access complications, pneumonia and pneumothorax requiring intervention. An independent Clinical Events Committee consisting of non-trial physicians reviewed and adjudicated the trial safety events. Safety during 12 months post-T0 was previously reported [17].

Reinterventions were evaluated for 24 months and defined as crossover to epicardial ablation, electric cardioversion, pharmaceutical cardioversion, pacemaker implant, implantable cardioverter defibrillator implant, repeat ablation, surgical ablation or other interventions.

### Statistical analysis

The intention-to-treat (ITT) population was defined as patients who had an attempted index procedure (first-stage epicardial ablation in the HA arm and first CA in the CA arm) and was the population used to evaluate safety. The modified ITT (mITT) population was defined as patients who had an attempted index procedure and had available effectiveness end-point data after the T0 visit and was the population used for effectiveness and reintervention analyses. All statistical tests use a 2-sided significance level of α = 0.05. The effectiveness end-point analysis tests the null hypothesis of no-treatment group difference, using a 5% 2-sided significance level and a Fisher’s exact test. The 95% 2-sided confidence interval and *P*-value are reported. SAS 9.3 (SAS, Cary, NC, USA) was used for statistical analysis.

## RESULTS

As previously published, the 102 patients in the HA arm and 52 patients in the CA arm (ITT population) had similar baseline profiles, with a mean age of 60.7 ± 7.9 years, 74.7% male, mean body mass index 29.7 ± 3.4 kg/m^2^, mean left atrial diameter 4.7 ± 0.4 cm and a normal left ventricular ejection fraction. The type of AF was persistent in 80.5% and long-standing persistent in 19.5%, with a mean 3.08 ± 3.36 years of AF.

**Figure 1:**
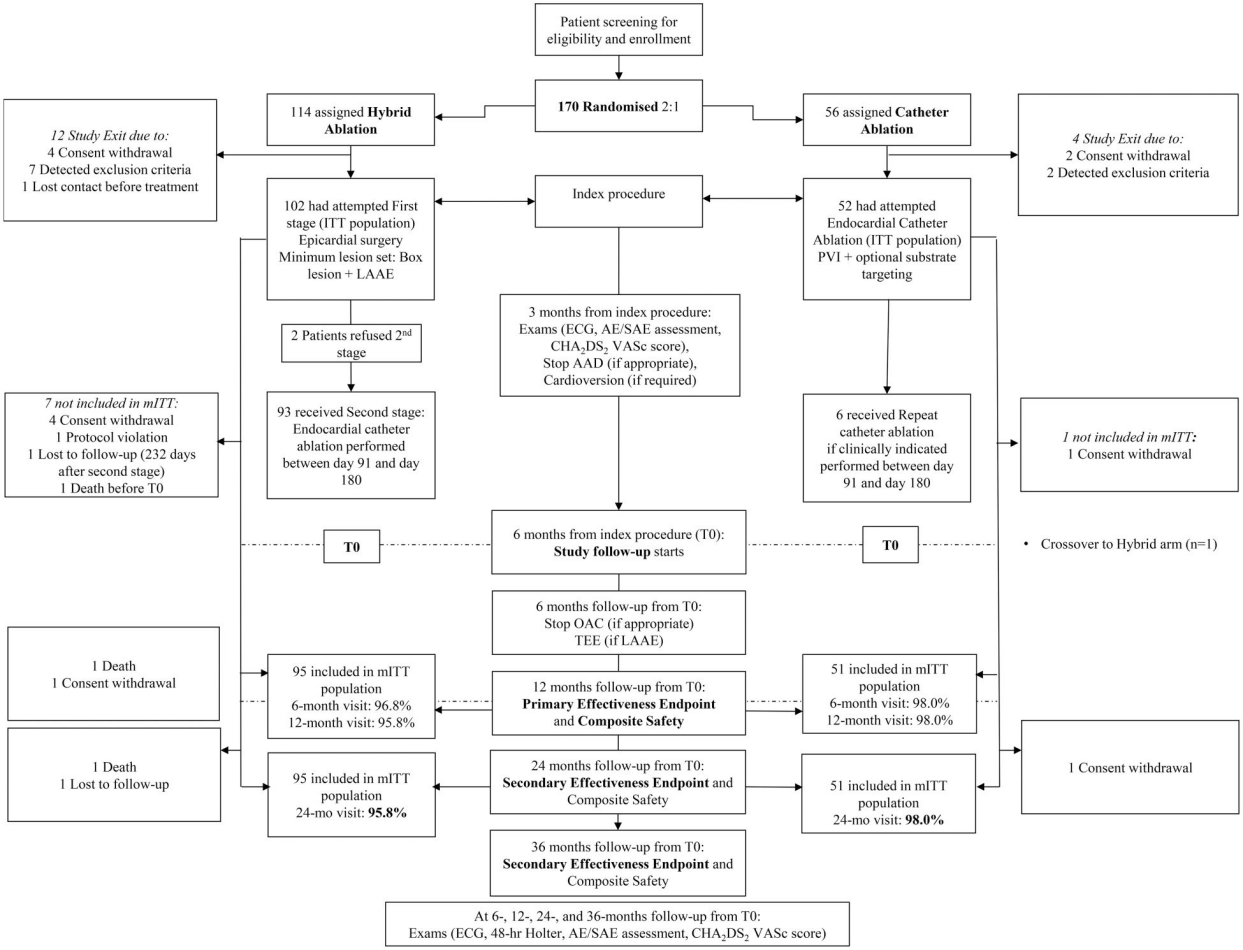
CONSORT diagram for CEASE-AF over the 24-month follow-up period. AAD: anti-arrhythmic; AE/SAE: adverse event/serious adverse event; CHA2DS2VASc: Congestive heart failure, Hypertension, Age ≥75 years (doubled), Diabetes mellitus, prior Stroke or TIA or thromboembolism (doubled), Vascular score; ECG: electrocardiogram; ITT: intention to treat; LAAE: left atrial appendage exclusion; mITT: modified intention to treat; PVI: pulmonary vein isolation; T0: index procedure plus 6 months; TEE: transesophageal echocardiography.

The mITT population comprised 146 patients (95 patients in the HA arm and 51 patients in the CA arm) (Fig. 1). Of these patients, 141 patients (91 in the HA and 50 in the CA arms) had a 24-month follow-up visit. In the HA arm, 2 patients died before the 24-month visit, 1 patient withdrew consent and 1 patient was lost to follow-up; in the CA arm, 1 patient withdrew consent.

### Rhythm outcomes during 24 months

**Figure 2:**
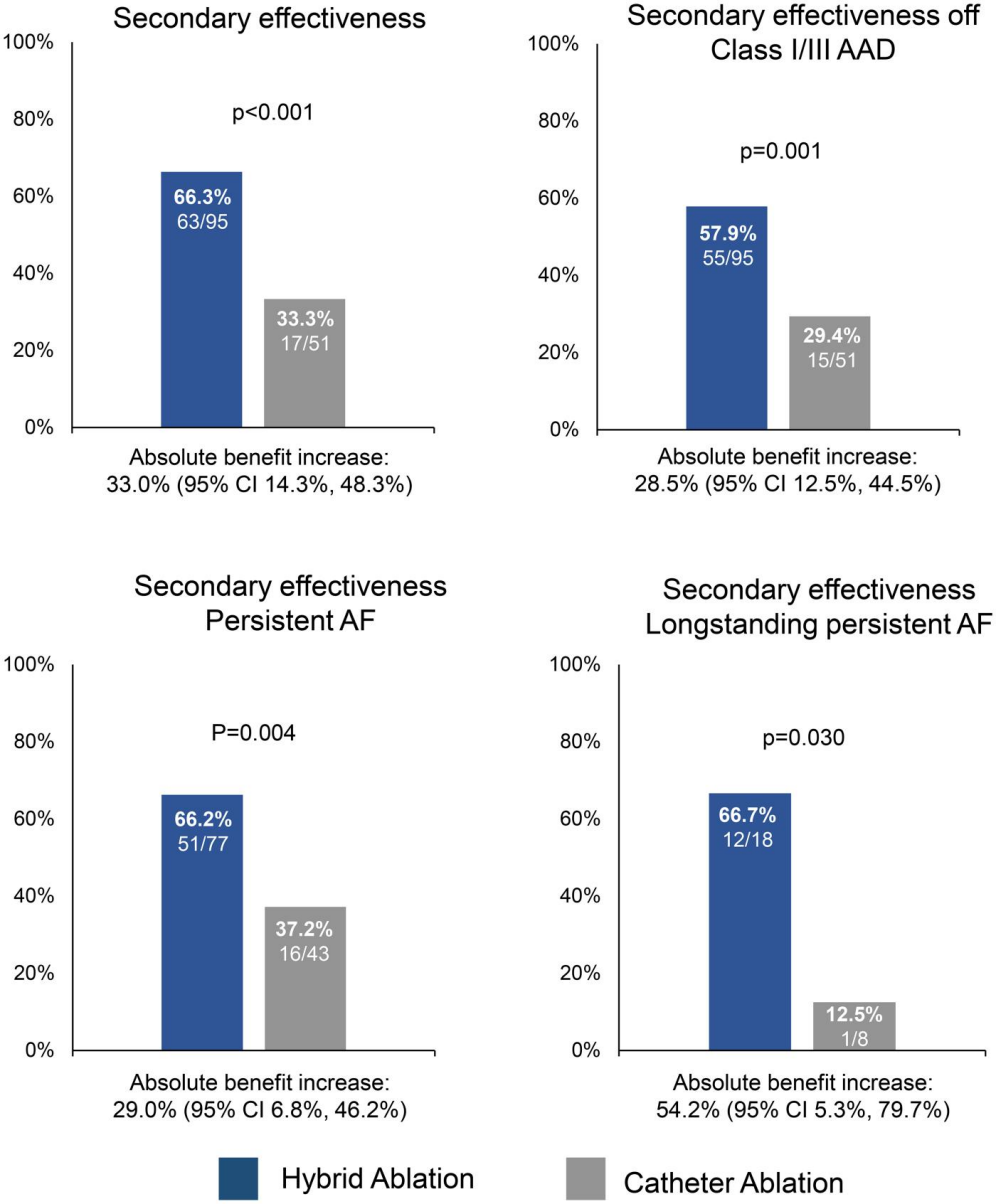
Freedom from atrial fibrillation, atrial flutter and atrial tachycardia off anti-arrhythmic drugs except those previously failed at doses not exceeding those previously failed, unless otherwise indicated, through 24 months in the hybrid and catheter ablation arms. AAD: anti-arrhythmic; AF: atrial fibrillation; CI: confidence interval.

In the mITT population, 143 patients (93 HA and 50 CA) had 24-month effectiveness data available. For the remaining patients, the last observation was carried forward. Effectiveness through 24 months post T0 was 66.3% (63/95) in the HA arm and 33.3% (17/51) in the CA arm [absolute difference 33.0% (95% confidence interval (CI), 14.3%, 48.3%; *P* < 0.001)] (Fig. 2). In subgroup analysis of patients with persistent AF at baseline, primary effectiveness was 66.2% (51/77) in the HA arm and 37.2% (16/43) in the CA arm [absolute different 29.0% (95% CI 6.8%, 46.2%; *P* = 0.004)]. In patients with long-standing persistent AF, primary effectiveness was 66.7% (12/18) in the HA arm and 12.5% (1/8) in the CA arm [absolute difference 54.2% (95% CI 5.3%, 79.7%; *P* = 0.030)]. Freedom from AF/AFL/AT off class I/III AADs was 57.9% (55/95) in the HA arm and 29.4% (15/51) in the CA arm [absolute difference 28.5% (95% CI 12.5%, 44.5%; *P* = 0.001)].

### Safety outcomes over 24 months

**Figure 3:**
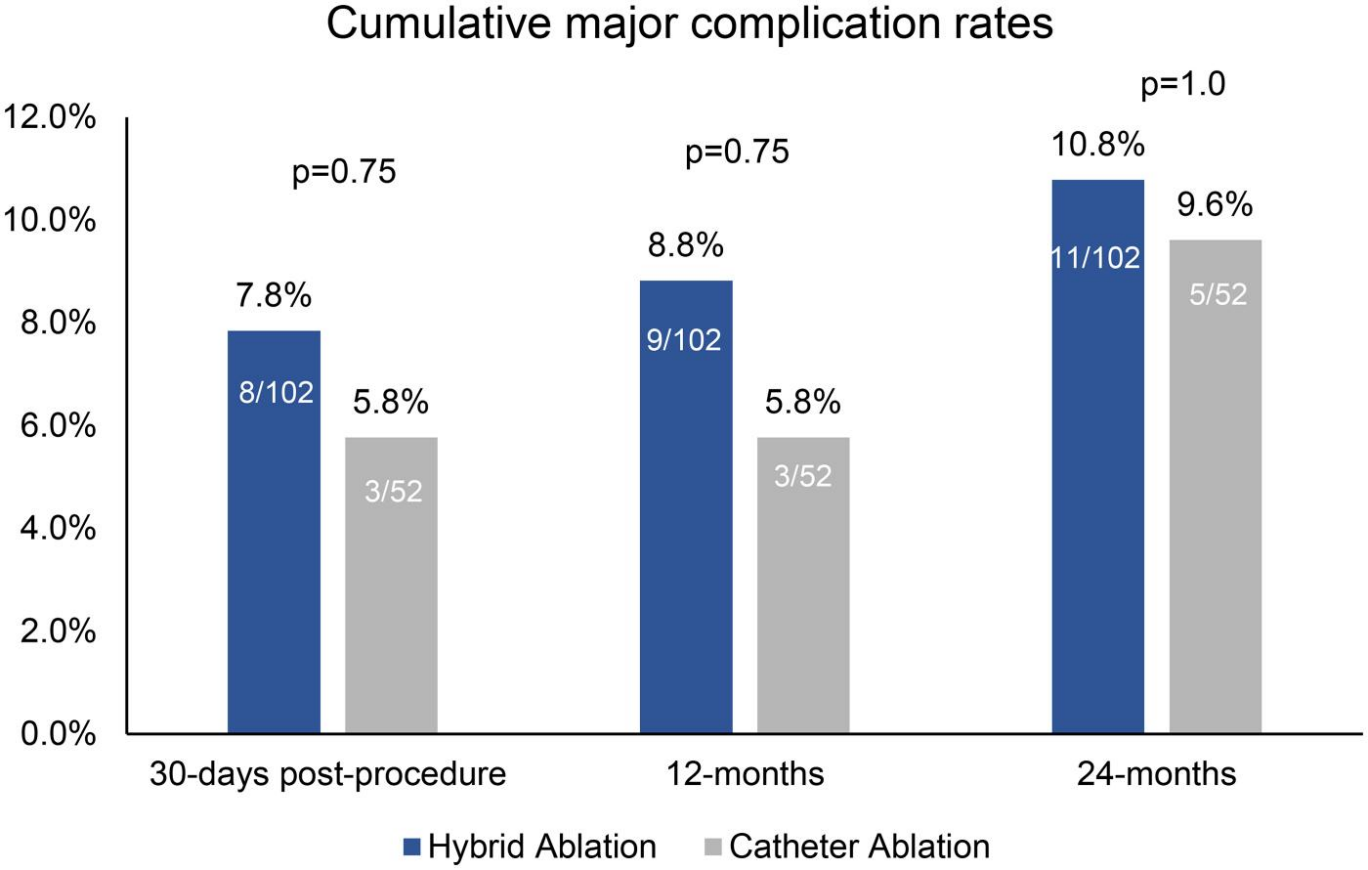
Cumulative major complications over 24 months in the intention-to-treat population.

In the ITT population, the proportions of patients who experienced major complications over 24 months after the index procedure were similar between arms, at 10.8% (11/102) in the HA arm and 9.6% (5/52) in the CA arm (*P* = 1.0) (Table 1). In the HA arm between 12 and 24 months from the index procedure, there was 1 cardiovascular death and 1 death of unknown cause. One patient died during sleep of undetermined cause; the other patient died of sudden cardiac arrest during vascular surgery on his lower limbs for symptomatic atherosclerosis. Pneumonia also occurred in a patient in the HA arm who had experienced an earlier major complication. In the CA arm, a patient experienced bleeding and vascular complications after transcatheter ablation, and another had community-acquired pneumonia. No new strokes or transient ischaemic attacks were reported. Cumulative major complication rates through 30 days post-index ablation and second stage/repeat ablation, through 12 months and through 24 months are shown in Fig. 3.

**Table 1:** Major complications over 24 months after the index procedure

Characteristic	Hybrid arm ITT population	Catheter arm ITT population	Total ITT population	Two-sided *P*-value
	(*n* = 102)	(*n* = 52)	(*N* = 154)	
Composite major complications within 730 days after index procedure	10.8% (11/102)	9.6% (5/52)	10.4% (16/154)	1.00
Type of major complication^a^				
Death (regardless of cause)	2.9% (3/102)	0.0% (0/52)	1.9% (3/154)	0.55
Stroke	1.0% (1/102)	0.0% (0/52)	0.6% (1/154)	1.00
Transient ischaemic attack	0.0% (0/102)	1.9% (1/52)	0.6% (1/154)	0.34
Myocardial infarction in the context of atrial fibrillation ablation	1.0% (1/102)	0.0% (0/52)	0.6% (1/154)	1.00
Pericarditis	1.0% (1/102)	1.9% (1/52)	1.3% (2/154)	1.00
Bleeding	1.0% (1/102)	1.9% (1/52)	1.3% (2/154)	1.00
Wound infection at surgical site or puncture sites requiring re-operation for wound debridement	0.0% (0/102)	0.0% (0/52)	0.0% (0/154)	
Major vascular access complications	1.0% (1/102)	1.9% (1/52)	1.3% (2/154)	1.00
Atrio-oesophageal fistula	0.0% (0/102)	0.0% (0/52)	0.0% (0/154)	
Oesophageal injury	0.0% (0/102)	0.0% (0/52)	0.0% (0/154)	
Permanent phrenic nerve paralysis	0.0% (0/102)	0.0% (0/52)	0.0% (0/154)	
Permanent pacemaker implant	1.0% (1/102)	0.0% (0/52)	0.6% (1/154)	1.00
Pulmonary vein stenosis	0.0% (0/102)	0.0% (0/52)	0.0% (0/154)	
Cardiac tamponade/cardiac perforation	0.0% (0/102)	1.9% (1/52)	0.6% (1/154)	0.34
Empyema	0.0% (0/102)	0.0% (0/52)	0.0% (0/154)	
Pneumothorax requiring intervention	1.0% (1/102)	0.0% (0/52)	0.6% (1/154)	1.00
Pneumonia	2.0% (2/102)	1.9% (1/52)	1.9% (3/154)	1.00
Other major complications	1.0% (1/102)	1.9% (1/52)	1.3% (2/154)	1.00

a Some patients experienced more than one major adverse event.

ITT: intention to treat.

### Reinterventions during 24 months

During 24 months in the mITT population, 18.9% (18/95) of patients in the HA arm and 52.9% (27/51) of patients in the CA arm had a reintervention procedure (defined in Methods) (*P* < 0.001) (Table 2). Cardioversions (electrical or pharmaceutical) were performed in 14.7% (14/95) of patients in the HA arm and in 29.4% (15/51) of patients in the CA arm (*P* = 0.034). The number of cardioversions ranged from 1 to 3 per patient in both arms. Two patients (2.1%) in the HA arm versus no patient in the CA arm received a permanent pacemaker as a repeat intervention (*P* = 0.34). One permanent pacemaker was implanted 485 days post-index procedure for atrioventricular block, and the other was implanted at 841 days post-index procedure for bradycardia with Morgagni-Adam-Stokes syndrom. The repeat ablation rate was 7.4% (7/95) in the HA arm and 37.3% (19/51) in the CA arm (*P* < 0.001). In addition, 9 patients (17.6%) in the CA arm crossed over to receive an epicardial ablation. After the epicardial ablation procedure, 6 patients remained in sinus rhythm at subsequent follow-up visits through 24 months. The outcomes of the other 3 patients who experienced arrhythmia following the crossover ablation are described in Supplementary Material, Table S1.

**Table 2:** Reinterventions over 24 months

Characteristic	Hybrid arm mITT population	Catheter arm mITT population	*P*-value
	(*n* = 95)	(*n* = 51)	
Number of interventions	27	56	
Patients with an intervention	18.9% (18/95)	52.9% (27/51)	<0.001
Number of interventions per patient			<0.001
0 events	81.1% (77/95)	47.1% (24/51)	
1 event	12.6% (12/95)	21.6% (11/51)	
2 events	3.2% (3/95)	15.7% (8/51)	
3 events	3.2% (3/95)	5.9% (3/51)	
4 events	0.0% (0/95)	9.8% (5/51)	
Mean number of interventions per patient	0.3 ± 0.7 (95)	1.1 ± 1.3 (51)	<0.001
Number of electrical/pharmaceutical cardioversions	17	24	
Patients with an electrical/pharmaceutical cardioversion	14.7% (14/95)	29.4% (15/51)	0.034
Number of electrical/pharmaceutical cardioversions per patient			0.022
0 events	85.3% (81/95)	70.6% (36/51)	
1 event	12.6% (12/95)	15.7% (8/51)	
2 events	1.1% (1/95)	9.8% (5/51)	
3 events	1.1% (1/95)	3.9% (2/51)	
Mean number of electrical/pharmaceutical cardioversions per patient	0.2 ± 0.5 (95)	0.5 ± 0.8 (51)	0.024
Number of implants of a permanent pacemaker	2	0	
Patients with an implant of a permanent pacemaker	2.1% (2/95)	0.0% (0/51)	0.54
Number of repeat ablations	8	23	
Patients with a repeat ablation	7.4% (7/95)	37.3% (19/51)	<0.001
Number of repeat ablations per patient			<0.001
0 events	92.6% (88/95)	62.7% (32/51)	
1 event	6.3% (6/95)	31.4% (16/51)	
2 events	1.1% (1/95)	3.9% (2/51)	
3 events	0.0% (0/95)	2.0% (1/51)	
Mean number of repeat ablations per patient	0.1 ± 0.3 (95)	0.5 ± 0.7 (51)	<0.001

mITT: modified intention to treat.

## DISCUSSION

Persistent AF with advanced left atrial pathology and long-standing persistent AF are recognized for their resistance to treatment with endocardial CA approaches. To date, no standard endocardial ablation strategy has been shown to effectively address advanced AF substrate resulting in consistent, durable clinical outcomes exceeding those of PVI. Therefore, the question remains whether standalone ablation efficacy is limited merely for these types of AF. The CEASE-AF trial showed that the addition of epicardial ablation and LAAE to endocardial CA resulted in significantly improved freedom from atrial arrhythmias with and without AADs through a 12-month follow-up period, with the positive benefit–risk ratio being supported by the absence of an increase in major complications [17]. A smaller randomized controlled trial HARTCAP-AF (Hybrid Versus Catheter Ablation in Persistent AF) also showed a significant benefit of HA and LAAE compared to CA including endocardial posterior wall isolation in all patients during a 12-month follow-up period [18]. In the present analysis, the CEASE-AF 24-month results demonstrate that the significant improvements in effectiveness after HA over CA are sustained through 24 months and that safety rates continued to be similar, though numerically higher, in the HA arm.

Until now, longer term outcomes of similar hybrid ablation techniques have been reported from single-centre observational studies. Pannone *et al.* [21] reported a 10-year experience with thoracoscopic hybrid ablation for treating AF either as a de novo or repeat procedure. At 12 months, freedom from atrial tachyarrhythmias without AADs was 76.7% and was 67.5% at 24 months. There were no significant differences in rates between patients who had a de novo ablation procedure, all of whom had persistent and long-standing persistent AF or a repeat procedure. Dunnington *et al.* [22] reported freedom from AF with and without AADs to be 88% and 80% through 12 months, respectively, and 79% and 75% through 24 months, respectively, after HA using RF bipolar pens and clamps and LAAE. Together, these studies confirm the durability of HA for preventing atrial arrhythmia recurrence shown in the CEASE-AF trial.

Repeat interventions including repeat ablation and cardioversions were more common in the CA arm than in the HA arm. Approximately half of the patients who experience AF recurrence after the initial CA will have repeat CA [23]. Repeat ablation rates after CA in patients with persistent and long-standing persistent AF are approximately 10% to 30%, and trials such as STAR-AF II (Substrate and Trigger Ablation for Reduction of Atrial Fibrillation Trial Part II) and CAPLA (Catheter Ablation for Persistent Atrial Fibrillation) suggest that endocardial ablation outside the PVs does not reduce repeat ablations in persistent and long-standing persistent AF [24, 25]. In this context, the repeat ablation rate observed in the CEASE-AF CA arm is not unexpected, particularly given the advanced AF population included. Through 24 months, 67% (34/51) of patients in the CA arm failed secondary effectiveness and 37% (19/51) had at least 1 repeat ablation; additionally, 17% (9/51) of patients crossed over to receive epicardial ablation. The repeat ablation rate in the HA, in which 33% (32/95) of patients failed secondary effectiveness, was significantly lower at 7.4% (7/95). The combined impact of arrhythmia recurrence, complications, AAD utilization and repeat interventions have the potential to impact patient quality of life, which is the subject of a separate analysis, and health care resource utilization, an analysis of which is warranted.

The PVs and left atrial posterior wall are key driver and substrate regions for AF; thus they are critical ablation targets in advanced AF. Successful acute isolation of these regions is important to restore sinus rhythm, and then durable isolation is important to sustain sinus rhythm. The epicardium and endocardium have been shown to have distinct activation patterns in AF, and an endocardial-only ablation approach may not be able to fully address the epicardial component [26]. Nakamura *et al.* [27] reported a 40% rate of epicardial reconnections after a first-pass endocardial roof and floor line to isolate the posterior wall after PVI. The epicardial reconnections were often present in the centre of the posterior wall and required multiple endocardial RF applications in multiple posterior wall segments to eliminate. Patients who have arrhythmia recurrences after endocardial ablation also have high reconnection rates at the PVs and posterior wall. In a 3-year follow-up of the CAPLA trial, 32% of patients who received endocardial PVI and posterior wall isolation required at least 1 redo ablation [25]. Among those patients, 75% had posterior wall reconnections and 55% had PV reconnections. A hybrid ablation approach that targets the epicardium and endocardium may facilitate durable, transmural lesions, resulting in higher and sustained effectiveness compared to an endocardial ablation approach.

## CONCLUSION

To date, CEASE-AF is the largest randomized controlled trial of HA with thoracoscopic LAAE compared to endocardial CA to report follow-up over 24 months. The absolute benefit of HA plus LAA exclusion to maintain freedom from atrial arrhythmias without new AADs or increased doses of previously failed AADs was 33% over CA over 24 months. This result extends the 32.4% absolute benefit observed through 12 months, underscoring the durability of HA during 24 months.

## Supplementary Material

ezaf146_Supplementary_Data

## Data Availability

The primary results of CEASE-AF will be made available on clinicaltrials.gov (NCT02695277) per FDAAA 801. Beginning 5 years after trial completion (last patient’s 12-month follow-up visit), partial data may be provided to qualified researchers through a direct request, including proposed research question(s), to MedAffairs@atricure.com, as determined by representatives of the Sponsor’s Scientific, Clinical, and Medical Affairs departments. Patient level data will not be made available.

## ABBREVIATIONS

AAD: Anti-arrhythmic drug
AF: Atrial fibrillation
AFL: Atrial flutter
AT: Atrial tachycardia
CI: Confidence interval
ITT: Intention to treat
LAAE: Left atrial appendage exclusion
mITT: Modified intention to treat
PV: Pulmonary vein
PVI: Pulmonary vein isolation
RF: Radiofrequency
