# Supplementary material for: Durable effectiveness and safety of hybrid ablation versus catheter ablation: 2-year results from the randomized CEASE-AF trial
Source: Eur J Cardiothorac Surg. 2025 Jul 25;67(7):ezaf146. doi: 10.1093/ejcts/ezaf146 (PMC12304665; doi:10.1093/ejcts/ezaf146)
Supplement: ezaf146_Supplementary_Data [file ezaf146_supplementary_data.zip › CEASE Suppl EJCTS.docx]

**Supplementary Table S1.** Outcomes of patients in the Catheter Ablation arm who crossed over to receive epicardial ablation and experienced arrhythmia

| **Patient** | **Rhythm, reintervention details** |
| --- | --- |
| 1 | Sinus rhythm at 12-month follow-up; Atrial flutter at 24-month follow-up |
| 2 | Supraventricular tachycardia at 12-month follow-up; endocardial ablation; sinus rhythm at 24-month follow-up |
| 3 | Electrical cardioversion 6 months after epicardial ablation, AF and bundle branch block at 12-month follow-up, and AF at 24-month follow-up |
